# Hæmorrhages in the Later Months of Pregnancy

**Published:** 1909-06-26

**Authors:** 


### Obstetrics.

### HÆMORRHAGES IN THE LATER MONTHS OF PREGNANCY.

The occurrence of bleeding from the uterus during the last two months of pregnancy is always a matter of serious import and may present great difficulties both in diagnosis and in treatment. Never a condition to be lightly passed over, it becomes one of the most serious import if the bleeding is due to placenta prævia, and the patient's life may depend upon correct diagnosis and prompt treatment. In general it may be said that hæmorrhage during the last two or three months of pregnancy is almost always caused by separation of the placenta from its site in a greater or lesser degree. Hæmorrhage caused in any other way is quite rare, and the bleeding then usually depends upon a new growth of the uterus. Partial separation of the placenta may occur coincidently with new growths of the uterus, and, of course, in no way caused by the growth.

We propose to confine ourselves here to cases of hæmorrhage due to partial separation of the placenta. The reason why a normally situated placenta begins to separate from the uterus is not always apparent. Although in some cases a fall or a blow may appear to be the cause, in others there is no accident or injury to which the lesion may be attributable. The only uterine lesions which seem likely to account for this are endometritis and subinvolution. In these conditions the endometrium, and therefore the decidua of pregnancy, is unduly vascular, containing thin walled dilated capillaries which look as if they would require only a very small alteration in the blood pressure to make them rupture. It is quite unlikely that any fœtal condition would dispose to separation of the placenta. On the other hand, we have no difficulty in understanding why a placenta situated in the lower uterine segment must separate as soon as the internal os begins to open up under the influence of uterine contractions. As the uterine contractions do commence to open the internal os as early as the sixth month in some instances, it is clear that a placenta prævia may give rise to hæmorrhage as early as this. Given that hæmorrhage more or less has occurred, the question at once arises whether the condition is accidental or unavoidable hæmorrhage, or, in other words, whether the bleeding is from a normally situated placenta or from a placenta prævia. To find this out a very careful examination is necessary. If the lower pole of the fœtus is the head, and it does not sink into the brim of the pelvis along with the lower uterine segment, there is at least a suspicion that the placenta fills the lower segment and prevents the fœtal head descending. It is practically an impossibility to palpate the placenta from the abdomen. By the vagina with a *closed cervix* a placenta prævia may be suspected if there is a great thickness of tissue between the examining finger and the presenting part, especially if this is more marked on one side. Naturally, if the cervix is open, a placenta prævia can actually be felt if any part of it is near enough to the os for the finger to reach it. If, however, the os is closed, and a diagnosis cannot be made, what is to be done next? If we gradually insinuate the finger through the os, we shall be able to detect the placenta if within reach, but if it is not in reach then the case may be one of partial separation of a normally situated placenta in which possibly no further hæmorrhage will occur, and yet our manipulations will cause labour to come on. Consequently we may be in a dilemma in these cases; in some we *must* terminate pregnancy (placenta prævia), in others judicious treatment may stave off labour and permit of the pregnancy going to full term, and yet if we bore the finger through the cervix for diagnostic purposes we shall probably terminate all alike. No definite law can be laid down in such cases; we must be guided by the hæmorrhage, its amount and its character, and whether it is repeated or not.

If a patient has repeated hæmorrhages in spite of rest in bed and sedatives, we are justified in dilating the cervix enough to admit the finger for diagnostic purposes. The gravity of the case may be put thus, that a woman with placenta prævia is never safe from the possibility of death from hæmorrhage until she is delivered and the uterus is under control. Supposing the absolute diagnosis of placenta prævia is made, what is best to be done? We can assume that the cervix will at least admit one finger, and there must have been uterine contractions to cause the separation of the placenta: consequently we have the two desiderata for a satisfactory delivery —uterine contractions and commencing dilatation of the os. What we have to provide for is complete dilatation without further loss of blood.

Without any doubt the safest way for the patient and the one which gives the child the best chance, is the introduction of Champetier de Ribe's hydrostatic bag. The objection to it is its perishable nature. This is a much more important criticism of it than the usual one—namely, that it displaces the presenting part. The bag, when not in use, must be kept partly full of water and blown out fairly tight with air. It must never be folded up. If blown out and kept in a moderately warm place, and occasionally taken out and handled, the bag will keep serviceable for a very long time. The superiority of the bag over version is manifest, for it can be put in through an os uteri which only admits one finger, it stops the hæmorrhage, it stimulates the uterus to contract. The half breech after version will also accomplish the latter two desiderata, but the patient may lose a fatal amount of blood whilst performing the version. Version cannot be done unless two fingers can be passed through the os, and even then is by no means easy for the man who does not encounter many obstetric emergencies. There is usually no difficulty whatever in putting the bag into the uterus, and when once there and filled the patient is practically safe from death from hæmorrhage. Moreover, any properly made bag can be boiled.

